# β-elemene promotes microglial M2-like polarization against ischemic stroke via AKT/mTOR signaling axis-mediated autophagy

**DOI:** 10.1186/s13020-024-00946-6

**Published:** 2024-06-15

**Authors:** Qiong Zhao, Lu Chen, Xin Zhang, Hua Yang, Yi Li, Ping Li

**Affiliations:** State Key Laboratory of Natural Medicines, China Pharmaceutical University, #639 Longmian Dadao, Nanjing, 211198 China

**Keywords:** β-elemene, Ischemic stroke, M1/M2-like polarization, AKT/mTOR, Autophagy

## Abstract

**Background:**

Resident microglia- and peripheric macrophage-mediated neuroinflammation plays a predominant role in the occurrence and development of ischemic stroke. Microglia undergo polarization to M1/M2-like phenotype under stress stimulation, which mediates intracellular inflammatory response. β-elemene is a natural sesquiterpene and possesses potent anti-inflammatory activity. This study aimed to investigate the anti-inflammatory efficacy and mechanism of β-elemene in ischemic stroke from the perspective of balancing microglia M1/M2-like polarization.

**Methods:**

The middle cerebral artery occlusion (MCAO) model and photothrombotic stroke model were established to explore the regulation effect of β-elemene on the cerebral ischemic injury. The LPS and IFN-γ stimulated BV-2 cells were used to demonstrate the anti-inflammatory effects and potential mechanism of β-elemene regulating M1/M2-like polarization in vitro.

**Results:**

In C57BL/6 J mice subjected to MCAO model and photothrombotic stroke model, β-elemene attenuated neurological deficit, reduced the infarction volume and neuroinflammation, thus improving ischemic stroke injury. β-elemene promoted the phenotype transformation of microglia from M1-like to M2-like, which prevented neurons from oxygen and glucose deprivation/reoxygenation (OGD/R) injury by inhibiting inflammatory factor release, thereby reducing neuronal apoptosis. Mechanically, β-elemene prevented the activation of TLR4/NF-κΒ and MAPK signaling pathway and increased AKT/mTOR mediated-autophagy, thereby promoting M2-like polarization of microglia.

**Conclusions:**

These results indicated that β-elemene improved cerebral ischemic injury and promoted the transformation of microglia phenotype from M1-like to M2-like, at least in part, through AKT/mTOR-mediated autophagy. This study demonstrated that β-elemene might serve as a promising drug for alleviating ischemic stroke injury.

**Supplementary Information:**

The online version contains supplementary material available at 10.1186/s13020-024-00946-6.

## Background

Ischemic stroke takes up 80% ~ 87% of total stroke cases, which represents the main factor inducing severe disability [1]. The occurrence of ischemic stroke triggers a chain of malignant cascades, including excitotoxicity, oxidase stress, inflammation, and apoptosis, causing brain cell death and neurological function loss [2, 3]. Currently, intravenous thrombolytic therapy is the most important measure to restore blood flow and improve ischemic stroke. Many drugs, including alteplase (rt-PA), urokinase, and teneplase, are commonly used in clinic. However, because of the narrow treatment window (t < 4.5 h) and reperfusion injury, their clinical efficacy is limited [4]. Therefore, there is a need to develop drugs alone or in combination with these drugs to expand therapeutic window in existing treatments, promote the therapeutic effect, and improve the prognosis of ischemic stroke.

Neuroinflammation has become the focus of attention in ischemic stroke. It is activated immediately and persists for weeks or even months after ischemic stroke, which implies the predominant effect on cerebral ischemic injury [5, 6]. The immune response is initiated once ischemic stroke occurs, which is achieved by damage-associated molecular patterns (DAMPs) in damaged neural cells for activating pattern recognition receptors in microglia. Subsequently, blood–brain barrier (BBB) collapses, causing peripheral immune cell activation and invasion. Notably, resident microglia and macrophages undergo a series of phenotypic changes depicted to be the functional dichotomy: classical (M1-like) as well as alternative (M2-like) activation [7]. In the M1-like phenotype, microglia/macrophages secrete pro-inflammatory mediators like IL-6, IL-1β, TNF-α, chemokines, and iNOS, which can damage neurons [8]. Under the M2-like phenotype, scavenger receptors and proangiogenic factors are expressed in microglia/macrophages, which is closely linked to neuroprotective effects. Microglial polarization is deemed to be crucial for the advancement of stroke. A switch of M1-like to M2-like phenotype of microglia blocks the mTOC1 pathway and improves stroke outcomes of middle cerebral artery occlusion (MCAO) mice [9]. In line with this, pro-inflammatory M1-like macrophage polarization exacerbates ischemic stroke in mice [10]. Moreover, serum IL-6 and TNF-α are abnormally elevated among stroke patients (< 24 h), and IL-6 level is positive correlation with stroke severity and poor outcomes [11]. Therefore, regulating the equilibration between M1-like and M2-like phenotype in microglia/macrophages may be an effective way to alleviate ischemic stroke injury.

Autophagy is the primary mechanism mediating the transport of cellular contents to lysosomes for degradation and recycling, which is essential for maintaining cell homeostasis [12]. It belongs to self-protection cellular process and can usually be activated by detrimental factors including ischemia and hypoxia [13]. Numerous studies have confirmed that autophagy is closely associated with multiple diseases, such as cardiovascular diseases [14], neurologic disorders [15], and endocrine system disease [16]. Activation of autophagy and Akt/CREB signaling pathway play a pivotal role in the neuroprotective effect of neonatal hypoxia–ischemia [17]. In microglia derived from MCAO mice, the generation of autophagosome and the increased autophagy process significantly inhibit neuroinflammation against ischemic brain injury [18]. Therefore, accelerating the process of autophagy may be a potential strategy for treating ischemic stroke.

As a sesquiterpene compound extracted from *Curcuma wenyujin*, β-elemene exerts anti-tumor and anti-inflammatory biological activities. The chemical structure of β-elemene is shown in Figure S1 of Additional file. Currently, β-elemene has been clinically utilized as an anti-tumor agent because of the superb anti-cancer effect and low toxicity [19]. Beyond the anti-tumor efficacy, β-elemene also alleviates heart failure by blocking lipids-induced inflammation [20], and inhibits cardiac inflammation via regulating JAK/STAT3-NF-κB pathway [21]. In sepsis-associated encephalopathy, β-elemene blocks RAC1/MLK3/p38 pathway to decrease proinflammatory cytokines production [22]. Although these studies verify that β-elemene possesses significant anti-inflammatory activity, its efficacy and implying mechanism in ischemic stroke are still unclear. In our work, β-elemene attenuated neurological deficit, reduced the infarction volume and neuroinflammation, thus improving ischemic stroke injury in C57BL/6 J mice subjected to MCAO model and photothrombotic stroke model. Moreover, β-elemene promoted the transformation of microglia from M1-like to M2-like phenotype in MCAO mice and LPS- and IFN-γ-induced BV-2 cells. Mechanically, β-elemene prevented the activation of TLR4/NF-κΒ and MAPK signaling pathway and increased AKT/mTOR mediated-autophagy, thereby promoting M2-like polarization of microglia. This study provides some proofs for applying β-elemene in clinic for the treatment of ischemic stroke.

## Materials and methods

### Reagents

Β-elemene (purity: 99.40%, 100,268-201903) was obtained from National Institutes for Food and Drug Control (Shanghai, China). Cell Counting Kit-8 (CCK-8) and BCA Protein Assay Kit were provided by Beyotime Biotechnology (Shanghai, China). LDH Assay Kit (CAT#A020-2-2) was bought from Nanjing Jiancheng Bioengineering Institute (Nanjing, China). 3-Methyladenine (CAT#S2767) was from Selleck Chemicals LLC (Houston, TX, USA). SC79 (HY-18749) and miltefosine (HY-13685) were purchased from MedChemExpress (MCE) (Monmouth Junction, NJ, USA). Trizol (CAT#R401-01), HiScript Reverse Transcriptase (CAT#R122-01), and ChamQ SYBR Color qPCR Master Mix (CAT#Q411-02) were acquired from Vazyme Biotech (Nanjing, China). DL‑3‑n‑butylphthalide (NBP) and edaravone were obtained from CSPC Pharmaceutical Co. Ltd. and China National Medicines Guorui Biomedical Technology Co., Ltd., respectively. Primary antibodies against β-actin (1:1000, CAT#81,115-1-RR), mTOR (1:1000, CAT#66,888-1-Ig), p-P65 (Ser536) (IF: 1: 100, WB: 1:1000, CAT#82,335-1-RR), MyD88 (1:1000, CAT#23,230-1AP), LC3 (1:1000, CAT#14,600-1-AP), AKT (1:1000, CAT#60,203-2-Ig), p-AKT (Ser 473) (1:1000, CAT# 80,455-1-RR), AMPK (1:1000, CAT#10,929-2-AP), and NeuN (1:1000, CAT#26,975-1-AP) were provided by Proteintech (Chicago, IL, USA), whereas F4/80 (1:100, CAT#ab6640), P65 (1:1000, CAT#ab32536), Iba-1 (IF: 1:100, WB: 1:1000, CAT#ab178846), P62/SQSTM1 (1:1000, CAT#ab109012), p-AMPK (Thr 183 + Thr 172) (1:1000, CAT#ab133448), and HIF-1α (1:1000, CAT#ab179483) were from Abcam (Cambridge, MA, USA). Anti-TLR4 (IF: 1: 100, WB: 1:1000, CAT#sc-293072) and anti-p-mTOR (Ser2448) (1:1000, CAT# sc-293133) were bought from Santa Cruz (Shanghai, China). Anti-GFAP (1:1000, CAT#45,946), anti-Beclin-1 (1:1000, CAT#3495), ERK1/2 (1:1000, CAT#4695), p-ERK1/2 (1:1000, CAT#9101), p38 (1:1000, CAT#8690), p-P38 (1:1000, CAT#4511) were provided by Cell Signaling Technology (Danfoss, MA, USA). JNK (1:1000, CAT#AF6318) and p-JNK (1:1000, CAT#AF3318) were bought from Affinity Biosciences (Cincinnati, OH, USA). Mouse IL-1β (CAT#EMC001b.96), IL-6 (CAT#EMC004.96), and TNF-α (CAT#EMC102a.96) enzyme-linked immunosorbent assay (ELISA) kits were bought from NeoBioscience (Beijing, China). Neural Tissue Dissociation Kit (P) (CAT#130-092-628) was from Miltenyi Biotec (Bergisch Gladbach, Germany). PE-anti-F4/80 (CAT#12-4801-80) and PC5.5-anti-CD86 (CAT#15-0862-82) were obtained from Elabscience (Houston, TX, USA). Fixed Viability Stain 780 (CAT#565,388), PE-Cy7-anti-CD11b (CAT#552,850), FITC-anti-CD45 (CAT#553,079), mouse BD Fc Block (CAT#553,141), APC-anti-CD206 (CAT#141,708), and Annexin V-PE/7-AAD apoptosis assay kit (CAT#559,763) were from BD Pharmingen (San Diego, CA, USA). Recombinant Murine IL-4 (CAT#214-4), IL-13 (CAT#210-13) and IFN-γ (CAT#315-05) proteins were provided by PeproTech (Rocky Hill, NJ, USA). TTC (CAT#T8877) were provided by Sigma Chemical (St. Louis, MO, USA). Enhanced ECL Chemiluminescent Substrate Kit (CAT#36222ES76) was acquired from Yeasen Biotech (Shanghai, China). Dulbecco’s Modified Eagle’s Medium (DMEM, CAT# KGM12800-500) was purchased from KeyGen Biotech (Nanjing, China).

## Cell culture and drug treatment in vitro

BV-2 cells and HT-22 cells were obtained from Shanghai Cell Bank of Chinese Academy of Sciences (Shanghai, China). The cells were cultivated in DMEM containing 10% FBS and 1% penicillin/streptomycin, and maintained in a sterile incubator containing 5% CO_2_ at a constant temperature of 37 ℃.

To induce polarization of M1-like phenotype microglia, 100 ng/mL LPS and 20 ng/mL IFN-γ were co-cultured with BV-2 cells for 24 h. To induce polarization of M2-like phenotype microglia, 20 ng/mL IL-4 and 20 ng/mL IL-13 were added to treat BV-2 cells for 24 h. For the OGD/R model, HT-22 cells were incubated in glucose-free DMEM medium in an anaerobic container containing 1.5% O_2_ for 4 h. Thereafter, a standard culture medium was replaced and cultured in the normoxic incubator for another 12 h.

## Cell viability

Cells (1 × 10^6^/mL) were inoculated into 96-well plates, and cultured with different concentrations of β-elemene. After 24 h, the cells were added to CCK8 solution (10 μL) for 30 min. The microplate reader was then utilized for measuring absorbance at 450 nm. Cell viability (%) = (OD_*sample*_—OD_*blank*_) / (OD_*control*_—OD_*blank*_) × 100%.

## Animals

The 8 ~  10 week-old male C57BL/6 J mice (22 ~  24 g) were offered by Model Animal Research Center (MARC) of Nanjing University (Nanjing, China). All animal care and experimental procedures gained approval from Animal Ethics Committee of China Pharmaceutical University (protocol code: 2021-12-006). Mice were fed under 22 ± 2 ℃, 60% humidity, 12 h/12 h dark/light cycle conditions, and allowed to drink water and eat food freely.

## Construction of MCAO model

The mice were anesthetized with isoflurane (3% induction and 1.5% maintenance) and immobilized in the supine position. Then, the left common carotid artery (CCA), external carotid artery (ECA), and internal carotid artery (ICA) were exposed. An incision was made at the ECA and the silicone coated suture (1800A, Guangzhou jialing) was inserted, pushed to the ICA, and wedged in cerebral arterial circle for obstructing the origin of middle cerebral artery (MCA). Knot the CCA to stop blood flow. The mice were kept for 60 min after occlusion and then the intraluminal filament was withdrawn. Sham control mice received identical anesthesia and artery exposure, except for the insertion of filament. The mice were kept under 37 ± 0.5 °C during the surgery and then returned to an individual cage when they recovered from anesthesia. The grouping and survival of mice during the experiment were summarized in Table S1 of the Additional file. After 24 h, the mice were sacrificed under deep anesthesia for cervical dislocation, and then the brain tissue was collected for follow-up experiments.

Animals were randomized as 7 groups: (i) Sham group that received 10% DMSO + 90% corn oil (i.p.); (ii) model group receiving 10% DMSO + 90% corn oil (i.p.); (iii) model group receiving 25 mg/kg β-elemene (i.p.); (iv) model group receiving 50 mg/kg β-elemene (i.p.); (v) model group receiving 100 mg/kg β-elemene (i.p.); (vi) model group receiving 50 mg/kg NBP (i.g.); (vii) model group receiving 10 mg/kg edaravone. According to the previous studies, mice were given 25, 50, 100 mg/kg β-elemene by intraperitoneal injection at 1 h before ischemia and 5 h after reperfusion, respectively [20, 23]. The NBP group were given 50 mg/kg NBP by intragastric administration at 10 min and 5 h after reperfusion, respectively [24]. The edaravone group was intraperitoneally injected at a dose of 10 mg/kg immediately after reperfusion [25].

The neurological function was evaluated using the 5-level 4-point method of Zea-Longa. 0 suggested the absence of neurological impairment symptom; 1 suggested failure in full extension of left forepaw, mild nerve function injury; 2 suggested contralateral forelimb pronation, shoulder adduction, medium nerve function injury; 3 suggested turning and tipping to the opposite side, severe neurological impairment; 4 suggested inability to walk autonomously and partial loss of consciousness.

## Photothrombotic stroke model

After anesthesia, mice were put into the stereotactic device (Wood Dale, IL, USA), followed by intraperitoneal injection of rose red (100 mg/kg) for 5 min. The skull was exposed through cutting the cortex, and the cold light source (11,500 lx) was placed at the right side of the skull 1.5 mm by a fiber bundle with a diameter of 2 mm for 15 min. The mice were kept under 37 °C to wake up. After the modeling, the mice were given β-elemene continuously for 3 days. The blood was collected, and then the mice were sacrificed to obtain brain tissue.

Animals were randomized as 5 groups: (i) Sham group that received 10% DMSO + 90% corn oil (i.p.); (ii) model group receiving 10% DMSO + 90% corn oil (i.p.); (iii) model group receiving 25 mg/kg β-elemene (i.p.); (iv) model group receiving 50 mg/kg β-elemene (i.p.); (v) model group receiving 100 mg/kg β-elemene (i.p.).

## TTC staining

The brain tissues were collected, prepared in 2 mm coronal slices, and then immersed in 2% TTC for 15 min and fixed in 4% paraformaldehyde overnight. The brain tissue sections were photographed and analyzed with Image J. The infarct ratio (%) was calculated by formula below: (contralateral hemispheric volume—ipsilateral hemispheric non-infarcted volume)/(contralateral hemispheric volume) × 100%.

## Reverse transcription-quantitative PCR (RT-qPCR)

The brain tissue was extracted on ice with Trizol reagent. The total RNA was converted to cDNA by HiScript Reverse Transcriptase Kit. RT-PCR amplification was performed with ChamQ SYBR Color qPCR Master Mix under the conditions below: 30 s under 95 °C; 10 s under 95 °C, 30 s under 60 °C for 45 cycles; 15 s under 95 °C, and 1 min under 60 °C. Relative mRNA levels were identified based on *Actb* and determined using 2^−ΔΔCT^ method. The primer sequences for target genes were shown in Additional file 1: Table S2.

## Western-blotting analysis

The brain tissue was added RIPA lysate containing protease/phosphatase inhibitors and homogenized on ice to extract total protein. The resultant lysate was subjected to 15 min centrifugation (12 000 rpm) under 4 ℃ to obtain supernatants. A BCA Protein Assay Kit was utilized for determining protein content. Proteins were separated by SDS-PAGE gel electrophoresis. Separated proteins were then electrophoretically transferred onto a nitrocellulose filter (NC) membrane, followed by 5% defatted milk for 2 h under ambient temperature. Primary antibodies were added and incubated for overnight at 4 ℃. After washing with TBST, the NC membranes were incubated with secondary antibody for 1 h under ambient temperature. Enhanced chemoluminescence (Shanghai, China) was adopted for protein band visualization, and Image J software was utilized for quantification.

## Flow cytometry

Neural Tissue Dissociation Kit was utilized to prepare single-cell brain tissue suspensions. The 30–70% Percoll gradient was applied for separating cells to obtain mononuclear cells. After washing with PBS, cell precipitation was resuspended by adding 100 μL PBS, followed by incubation with Rat Anti-Mouse CD16/CD32 (Mouse BD Fc Block) (2.4G2) at 4 ℃ for 20 min to block nonspecific Fc receptor binding, and then labeling with Fixed Viability Stain 780, PE-Cy7-anti-CD11b, FITC-anti-CD45, PC5.5-anti-CD86, and APC-anti-CD206 on ice in dark for 20 min. For BV-2 cell staining, Fixed Viability Stain 780, PE-anti-F4/80, PC5.5-anti-CD86, and APC-anti-CD206 were added to label cells. For detection the apoptosis level of HT-22 cells, Annexin V-PE/7-AAD apoptosis assay kit was used according to the specific protocols. Fluorescence was detected by Multicolor flow cytometry (CytoFLEX S). FlowJo software (FlowJo LLC, Ashland, OR, USA) was applied for result analysis.

### ELISA

Blood from sham and model mice was centrifugated at 3000 rpm for 15 min to separate serum. Cell culture media was acquired, followed by centrifugation at 3000 rpm for 15 min to collect the supernatant for subsequent analysis. Corresponding ELISA Kit was utilized to detect the levels of TNF-α, IL-6, and IL-1β following the protocols of the manufacturer.

## Immunofluorescence

The brain tissue of mice was processed by 4% paraformaldehyde fixation and paraffin embedding, and then cut into a thickness of 10 μm. The brain slices were dehydrated with ethanol, blocked with goat serum, followed by primary antibody incubation at 4 ℃ overnight, and secondary antibody incubation at room temperature. Images were obtained using laser confocal photography (Olympus FV3000).

## H&E staining

The heart, liver, and kidney tissues were subjected to 4% paraformaldehyde fixation, dehydration, paraffin embedding, and preparation into 10 μm sections for H&E staining. An inverted microscope (OLYM-PUS, IXplore Standard, Tokyo, Japan) was adopted for image observation, and the Nikon DS-U3 software was applied in image capture.

## Conditioned media

BV-2 cells were treated with 100 ng/mL LPS and 20 ng/mL IFN-γ for 24 h. β-elemene (40 μM or 80 μM) was incubated for 6 h. Thereafter, freshly DMEM medium was replaced for 24 h and obtained the supernatant as the CM.

## LDH analysis

HT-22 cells were cultured into 96-well plates for OGD/R or CM treatment. The supernatant was collected to measure LDH level with an LDH Assay Kit.

## Statistical analysis

Results are represented by mean ± SEM (In vitro experiments: n = 5, n represents independent experiments; In vivo experiments: n ≥ 5, n represents the number of mice). Statistical analysis was completed with Prism 8.0 software (GraphPad, San Diego, CA, USA). The data among multiple groups were compared using one-way ANOVA test followed by Tukey’s test. Each experiment was blinded and randomized for avoiding unwanted bias and generating equal-sized groups. For in vivo experiments, the mice with MCAO operation dying on the day and that did not reach the experimental endpoint were excluded from the analysis. *p* < 0.05 represented significant difference.

## Results

### β-elemene improved ischemic stroke in MCAO mice

**Fig. 1:**
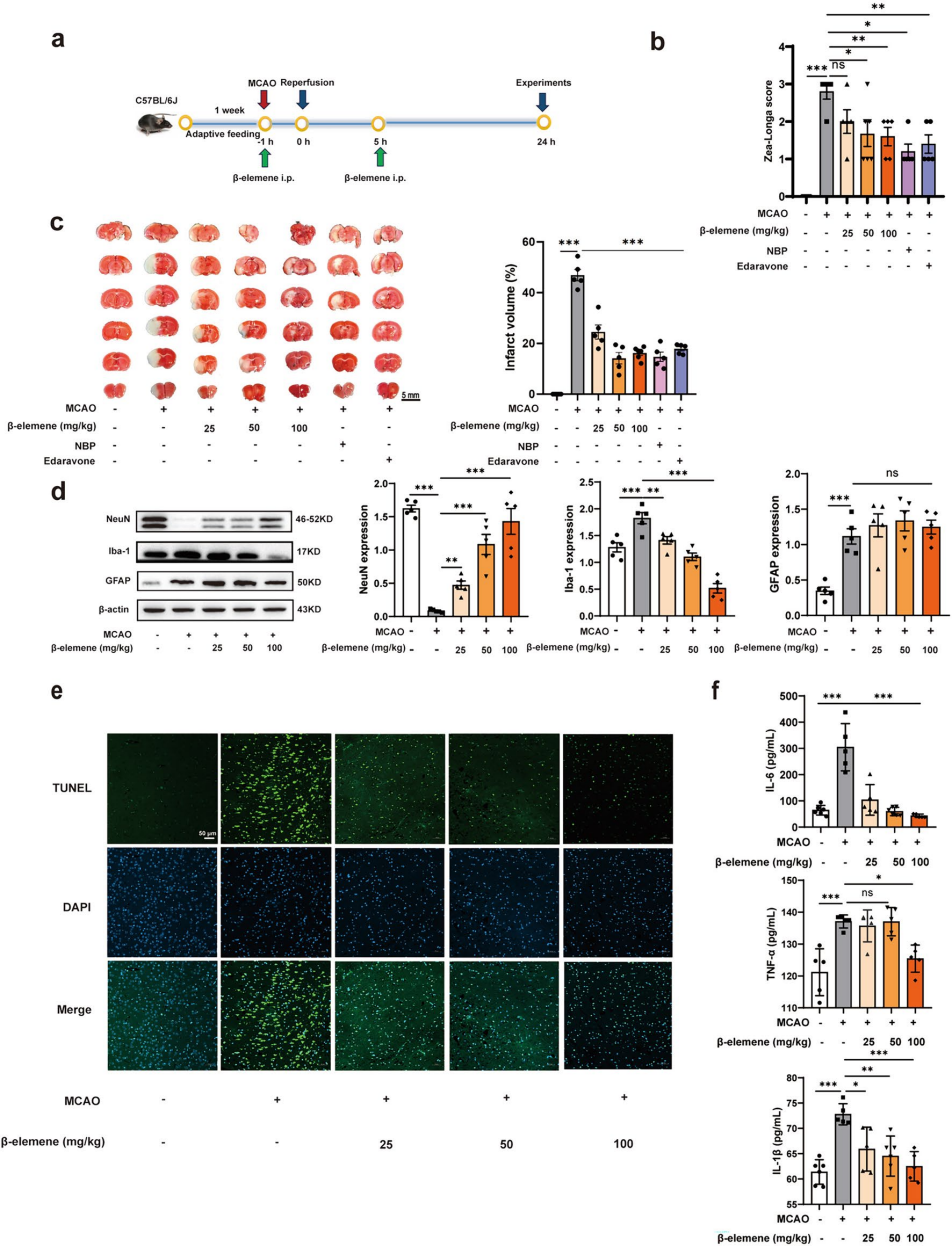
β-elemene improved ischemic stroke in MCAO mice. **a **Schematic of experimental timeline; **b** Neurological deficit of mice was assessed using the 5-level 4-point method of Zea-Longa; **c** 2,3,5-triphenyltetrazolium chloride (TTC) -stained brain sections after MCAO model and quantification of total infarct volume, scale bar = 5 mm; **d** NeuN, Iba-1 and GFAP protein expression within ischemic hemisphere of MCAO mice; **e** TUNEL (green) staining on brain slices, scale bar = 100 μm; **f** IL-6, TNF-α, and IL-1β levels within blood of MCAO mice. Results were indicated by means ± SEM (n ≥ 5, n represents the numbers of mice in each group). *p < 0.05, **p < 0.01, ***p < 0.001. *p-*values are analyzed using one-way ANOVA followed by Tukey’s test. ns, no significant difference

To evaluate the neuroprotection effect of β-elemene on ischemic stroke, an MCAO mouse model was established. It has been reported that estrogen exerts protective effect on cerebral ischemia injury, so male C57BL/6 J mice were chosen to establish the MCAO model [26]. A schematic depicting of mouse modeling and sample collection was shown in Fig. 1a. At first, the MCAO model was verified by monitoring cerebral blood flow (CBF) before MCAO, after MCAO, and after reperfusion (Additional file: Figure S2a). Compared with the sham group, the CBF of the MCAO group decreased by about 75% after ischemia and recovered to 60% after reperfusion, indicating successful ischemia/reperfusion surgery. As expected, MCAO operation led to significant neurological deficits and abnormally enlarged cerebral infarction volume, whereas β-elemene reversed these alterations in MCAO mice (Fig. 1b, c). NBP and edaravone are commonly used in the treatment of cerebral ischemia. As expected, they significantly improved neurological deficits and reduced infarct volume in MCAO mice. Next, we explored the regulatory effects of β-elemene on neurons, microglia and astrocytes by detecting the characteristic markers NeuN, Iba-1 and GFAP expression, respectively. As shown in Fig. 1d, β-elemene dose-dependently increased NeuN expression but decreased Iba-1 expression in the ischemic hemisphere of MCAO mice. In contrast, β-elemene displayed less effect on the expression of GFAP, suggesting that it had beneficial effects on neurons and microglia. TUNEL staining showed that MCAO operation greatly increased the apoptosis of cells in peri-infarct area characterized by enhanced green fluorescence, while treatment with β-elemene reversed this pathological change (Fig. 1e). In accordance with this, the NeuN staining assay on the hippocampus region showed that β-elemene protected neurons from apoptosis after MCAO surgery (Additional file: Figure S2b). Meanwhile, a tendency towards the lower IL-6, TNF-α, and IL-1β levels was also observed within blood of mice after administration of β-elemene (Fig. 1f). Hematoxylin–eosin Staining (H&E) staining assay indicated that no marked pathological damage was observed in heart, liver, and kidney in MCAO mice (Additional file: Figure S2c). Meanwhile, we evaluated the neuroprotection effect of β-elemene on the photothrombotic stroke model, and observed that β-elemene increased cortex CBF and decreased cerebral infarct volume of model group (Additional file: Figure S3a-S3b). It also suppressed the expression of Iba-1 around cerebral infarction, along with the decreased IL-6 levels in peripheral blood (Additional file: Figure S3c-S3d). Collectively, these data suggested that β-elemene ameliorated ischemic stroke injury.

## β-elemene promoted the switch of microglia from M1-like to M2-like phenotype in MCAO mice

**Fig. 2:**
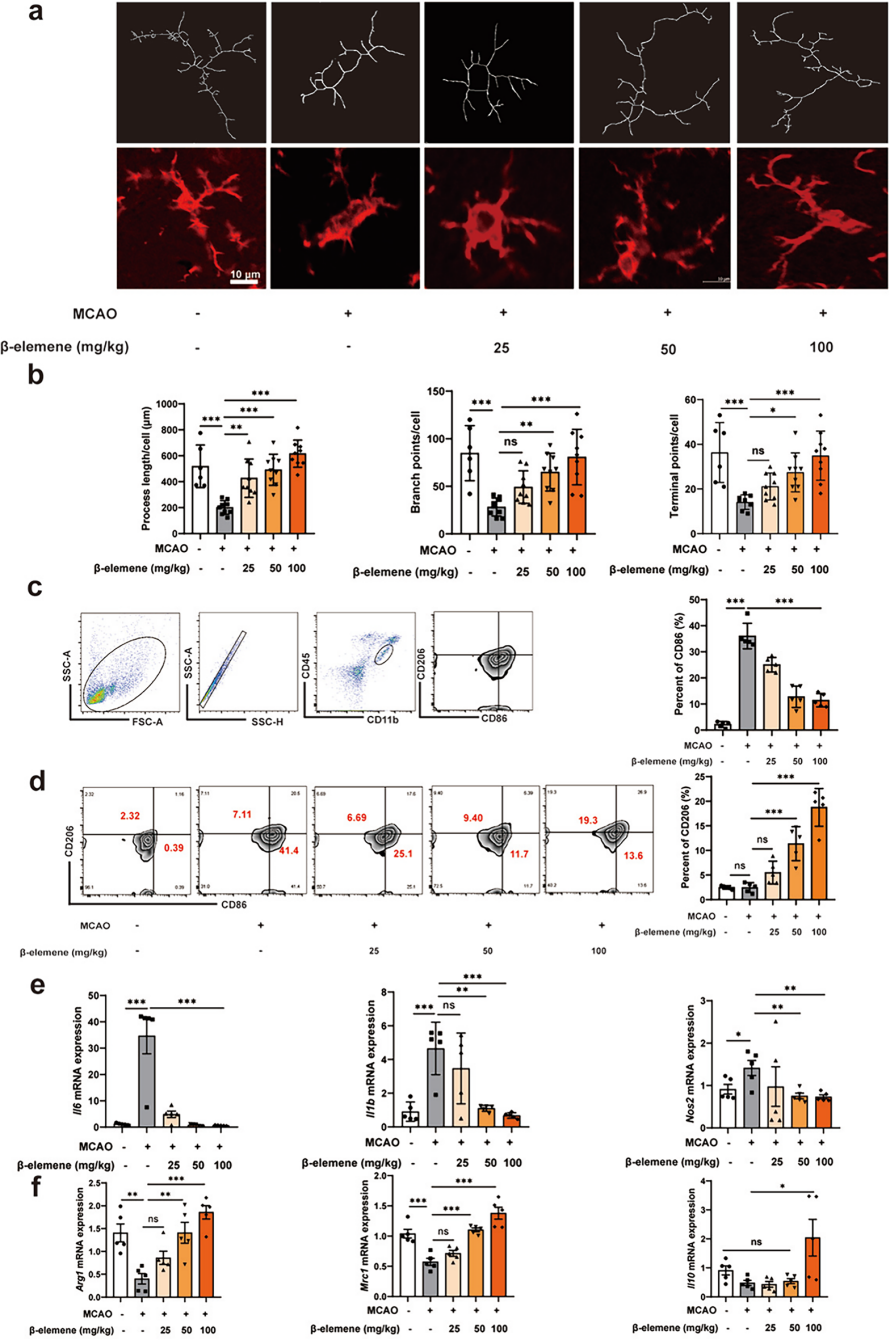
β-elemene promoted the transformation of microglia from M1-like to M2-like phenotype in MCAO mice. **a** Typical image showing Iba-1 expression and Z-stack projection for microglia within peri-infarct in ischemic hemisphere, scale bar = 10 μm; **b** microglial skeletal analysis on total length, branch point and terminal point numbers in MCAO mice; **c **gating strategy used to identify infiltrating and resident microglia after MCAO model; **d** proportions of M1-like and M2-like phenotype microglia in the ischemic hemisphere; **e, f** M1-like and M2-like phenotype-associated genes levels in the ischemic hemisphere. Results were represented by means ± SEM (n = 5). *p < 0.05, **p < 0.01, ***p < 0.001. *p*-values were analyzed using one-way ANOVA followed by Tukey’s test. ns, no significant difference

The microglia inherent in the brain tissue and peripherally derived macrophages can be activated rapidly after ischemic stroke. Activated microglia exhibited morphological changes and polarized into M1-like or M2-like phenotypes. Thus, we determined the morphology of microglia in peri-infarct area using the confocal Z-stack projections. Microglial skeletal analysis indicated that not only the total length of microglial branches of MCAO mice decreased relative to the sham mice, but also the branch and terminal point numbers per cell were reduced, suggesting that microglia were activated. Whereas these morphological changes of microglia were rescued after β-elemene treatment in MCAO mice (Fig. 2a, b). Consistently, pro-inflammatory microglia (CD86^+^CD206^−^) were significantly increased after MCAO operation by flow cytometry analysis. After administration of β-elemene, the proportion of the M1-like phenotype (CD86^+^CD206^−^) decreased while the M2-like phenotype (CD206^+^CD86^−^) increased (Fig. 2c, d). Meanwhile, β-elemene greatly down-regulated M1-like phenotype-associated genes *Il6, Il1b*, and *Nos2* mRNA expression while up-regulated M2-like phenotype-associated genes *Arg1*, *Mrc1*, and *Il10* mRNA expression in the ischemic hemisphere of MCAO mice (Fig. 2e, f). The above results collectively demonstrated that β-elemene repressed the neuroinflammation through promoting the switch of M1-like to M2-like phenotype microglia in MCAO mice.

## β-elemene promoted microglia M2 polarization in vitro

**Fig. 3:**
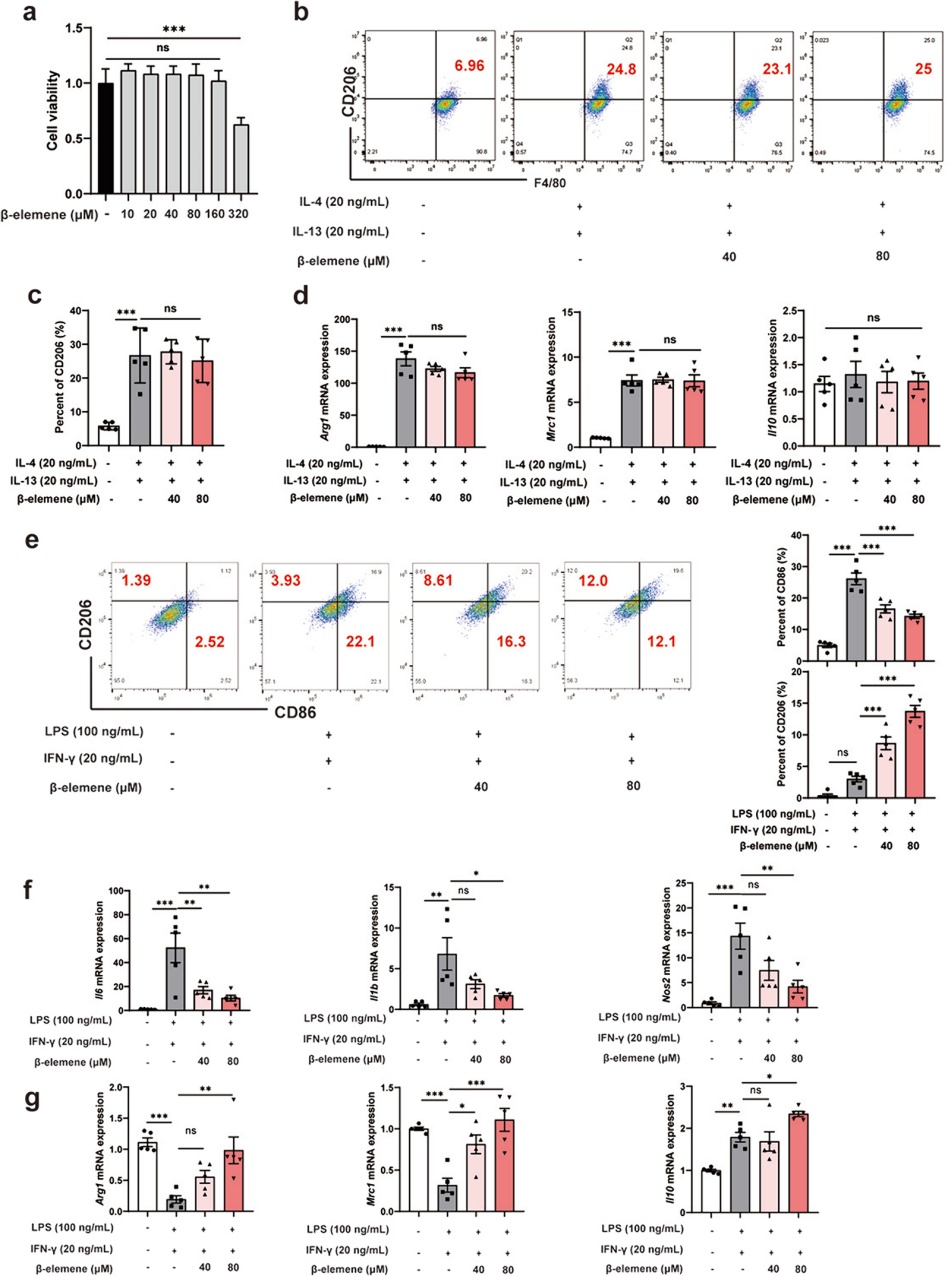
β-elemene promoted microglial M2-like polarization in vitro. **a** The BV-2 cell viability was determined with β-elemene at indicated doses for 24 h; **b–d** β-elemene (40, 80 μM) was added to BV-2 cells with 20 ng/mL IL-4 and 20 ng/mL IL-13 for 24 h. Typical image showing M2-like microglial proportion (**b, c**) and M2-like phenotype-associated gene levels (**d**); **e–g** BV-2 cells were incubated with β-elemene (40, 80 μM) when exposed to 100 ng/mL LPS and 20 ng/mL IFN-γ for 24 h. (**e**) Typical image showing M1/M2-like microglial proportion; **f, g** M1-like and M2-like phenotype-associated genes levels were analyzed. Results were represented by means ± SEM (n = 5). *p < 0.05, **p < 0.01, ***p < 0.001. *p*-values were analyzed through one-way ANOVA followed by Tukey’s test. ns, no significant difference

Subsequently, the microglia polarization model in vitro was established to investigate the role of β-elemene in regulating M1/M2-like paradigm. BV-2 cell is a type of microglia derived from mice, which retains microglia morphological and functional characteristics and is widely used in neuroscience research. The CCK-8 results confirmed the toxicity of β-elemene to BV-2 cells at high-dose (> 160 μM) (Fig. 3a). Therefore, we chose a lower dose for following study. F4/80 is cell surface glycoprotein, which has been widely used as unique marker of murine microglia/macrophages. Cells were treated with IL-4 and IL-13 for promoting the production of anti-inflammatory factors and driving microglia to M2 polarization (F4/80^+^/CD206^+^). As revealed by flow cytometry analysis, IL-4 and IL-13 stimulation significantly increased M2 polarization of microglia, and β-elemene treatment exerted few influences on this result (Fig. 3b, c). Correspondingly, β-elemene also did not alter M2-like phenotype-associated genes *Arg1*, *Mrc1*, and *Il10* expression upon IL-4 and IL-13 induction (Fig. 3d). However, CD86^+^CD206^−^ (M1-like phenotype) microglial proportion dose-dependently declined, and CD206^+^CD86^−^ (M2-like phenotype) microglia proportion increased in LPS- and IFN-γ-mediated BV-2 cells after β-elemene treatment, which showed the role of β-elemene in promoting the transformation of microglia from M1-like to M2-like phenotype (Fig. 3e). In support, β-elemene significantly decreased M1-like associated gene levels but increased M2-like associated-gene expression in LPS-and IFN-γ-mediated BV-2 cells (Fig. 3f, g). Taken together, β-elemene promoted the transformation of microglia from M1-like to M2-like phenotype for correcting the imbalance between these two phenotypes.

## β-elemene exerted neuroprotective function by inhibiting the activation of microglia

**Fig. 4:**
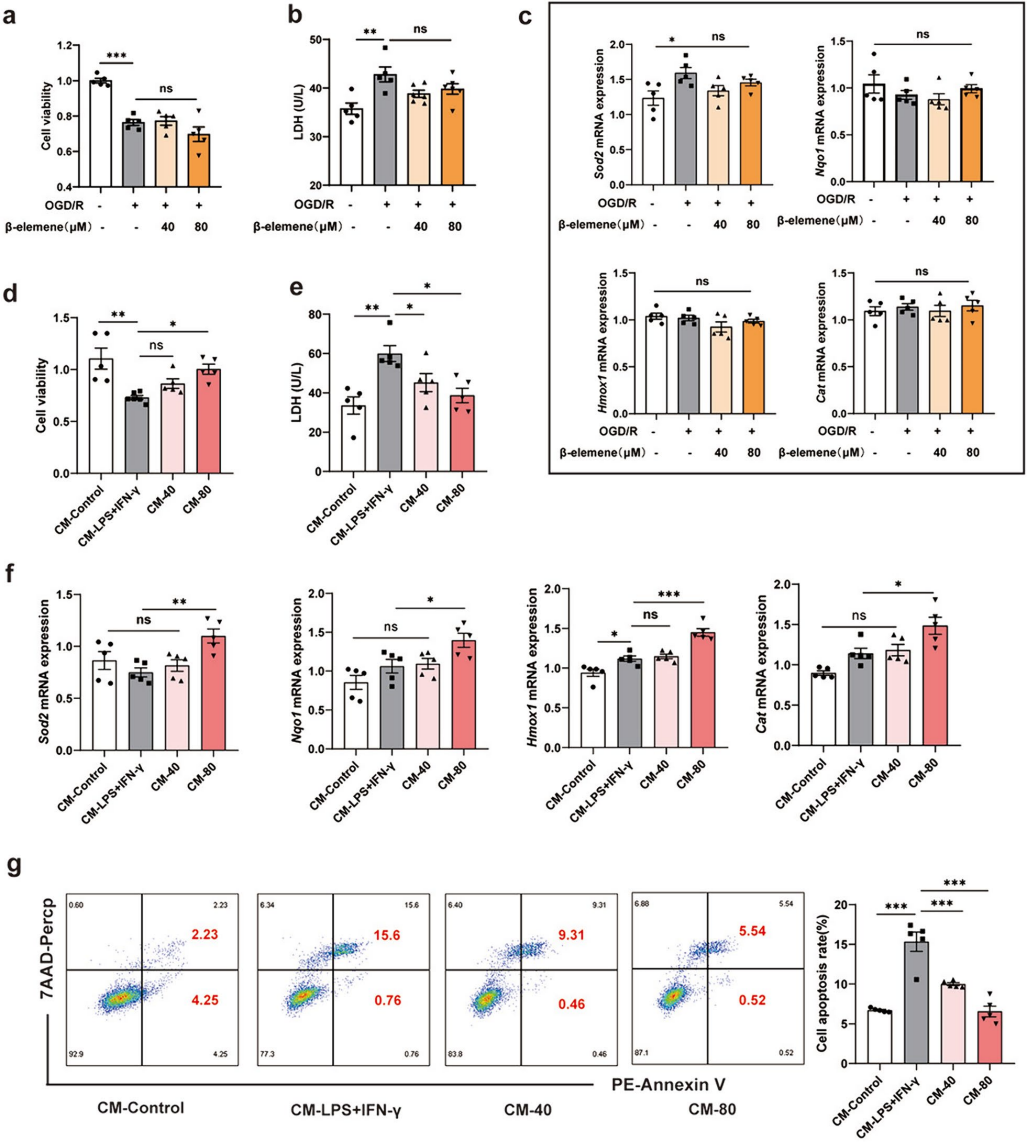
β-elemene exerted neuroprotective function by inhibiting the activation of microglia. **a** HT-22 cell viability was determined with OGD 4 h/R12 h stimulation; **b** the supernatant from HT-22 cells was obtained and LDH levels were assessed; **c** the mRNA of antioxidant-associated genes was detected in OGD/R-mediated HT-22 cells; **d–g** supernatant from BV-2 cells as a CM to stimulate HT-22 cells. The viability of HT-22 cells (**d**), the release of LDH (**e**), the mRNA of antioxidant-associated genes (**f**), and the apoptosis of HT-22 cells were analyzed (**g**). Results were represented by means ± SEM (n = 5). *p < 0.05, **p < 0.01, ***p < 0.001. *p*-values were analyzed through one-way ANOVA followed by Tukey’s test. ns, no significant difference

Sequentially, we investigated the role of β-elemene in regulating neurons, and a neuronal damage model based on OGD/R was established. HT-22 cell is a mouse hippocampal neuron cell line derived from HT4 cell line, which is a valuable model for studies of function of neuronal cells in vitro. Interestingly, treatment with β-elemene (40 μM, 80 μM) could not mitigate the damage on HT-22 cells caused by OGD/R (Fig. 4a, b). Furthermore, we examined the mRNA levels of antioxidant-associated genes (*Sod2*, *Nqo1*, *Hmox1*, and *Cat*) in HT-22 cells and discovered that β-elemene made no difference to the expression levels of these genes (Fig. 4c). These results confirmed that β-elemene could not exert protective effects on neurons directly. Therefore, it was speculated that neuroprotective effect of β-elemene was probably associated with the polarization of M1/M2-like phenotype microglia. We collected the supernatant of LPS-and IFN-γ-treated BV-2 cells as CM to stimulate HT-22 cells. As shown in Fig. 4d, e, β-elemene-treated CM remarkably increased the viability of HT-22 cell and reduced the release of Lactate Dehydrogenase (LDH). Consistently, the mRNA levels of antioxidant-associated genes (*Sod2*, *Nqo1*, *Hmox1*, and *Cat*) in HT-22 cells were significantly increased after β-elemene-treated CM treatment (Fig. 4f). Flow cytometry analysis also found that the apoptosis ratio of neurons was reduced with the β-elemene-treated CM treatment (Fig. 4g). These data revealed that β-elemene exerted indirect neuroprotective function by inhibiting the activation of inflammatory M1-like phenotype microglia.

## β-elemene prevented the activation of TLR4/NF-κΒ and MAPK signaling pathways in microglia

**Fig. 5:**
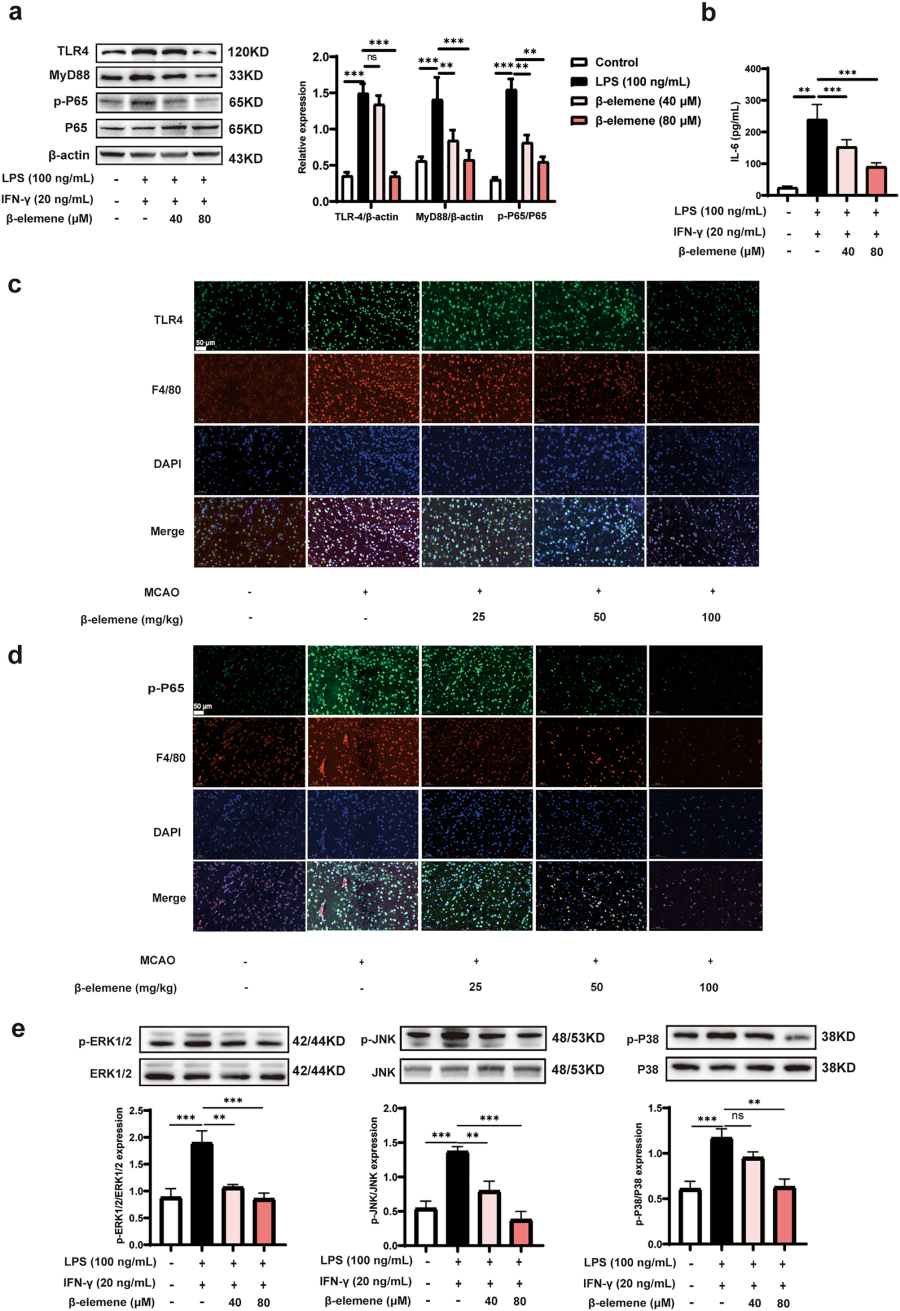
β-elemene prevented the activation of TLR4/NF-κΒ and MAPK signalling pathways in microglia. **a** TLR4, MyD88, p-P65, and P65 protein levels in LPS- and IFN-γ-treated BV-2 cells; **b** the level of IL-6 in LPS- and IFN-γ-treated BV-2 cells; **c, d** TLR4 and p-P65 protein levels within microglia in the peri-infarct area of the MCAO mice, scale bar = 50 μm. **e** p-ERK1/2/ERK1/2, p-JNK/JNK and p-P38/P38 protein levels in LPS- and IFN-γ-treated BV-2 cells; Results were indicated by means ± SEM (n = 5). **p < 0.01, ***p < 0.001. *p*-values are analyzed through one-way ANOVA followed by Tukey’s test. ns, no significant difference

It has been reported that TLR4/NF-κΒ and MAPK signaling pathways were activated in M1-like phenotype microglia [27, 28]. Thus, we first explored the regulation of β-elemene on TLR4/NF-κΒ signaling pathway. As expected, β-elemene prevented the expression of TLR4 and MyD88, and reduced P65 phosphorylation in LPS-and IFN-γ-treated BV-2 cells (Fig. 5a). Consequently, β-elemene dramatically reduced the IL-6 production at the concentrations of 40 μM and 80 μM (Fig. 5b). In line with this, immunofluorescence staining observed the strong activation of F4/80-positive microglia/macrophages, and up-regulated the expression of TLR4 and p-P65 in the peri-infarct area of the MCAO mice, whereas β-elemene treatment observably attenuated the activation of TLR4/NF-κΒ signaling pathway (Fig. 5c, d). In addition, we also investigated the effects of β-elemene on MAPK signalling pathway, including the expression of p-ERK1/2/ERK1/2, p-JNK/JNK and p-P38/P38. The results showed that β-elemene significantly inhibited the phosphorylation of ERK1/2, JNK and P38, which indicated the inhibitory effect on MAPK pathway activation (Fig. 5e). These results supported that β-elemene prevented the activation of TLR4/NF-κΒ and MAPK signaling pathways in M1-like phenotype microglia.

## β-elemene promoted autophagy to enhance microglia M2-like polarization

**Fig. 6:**
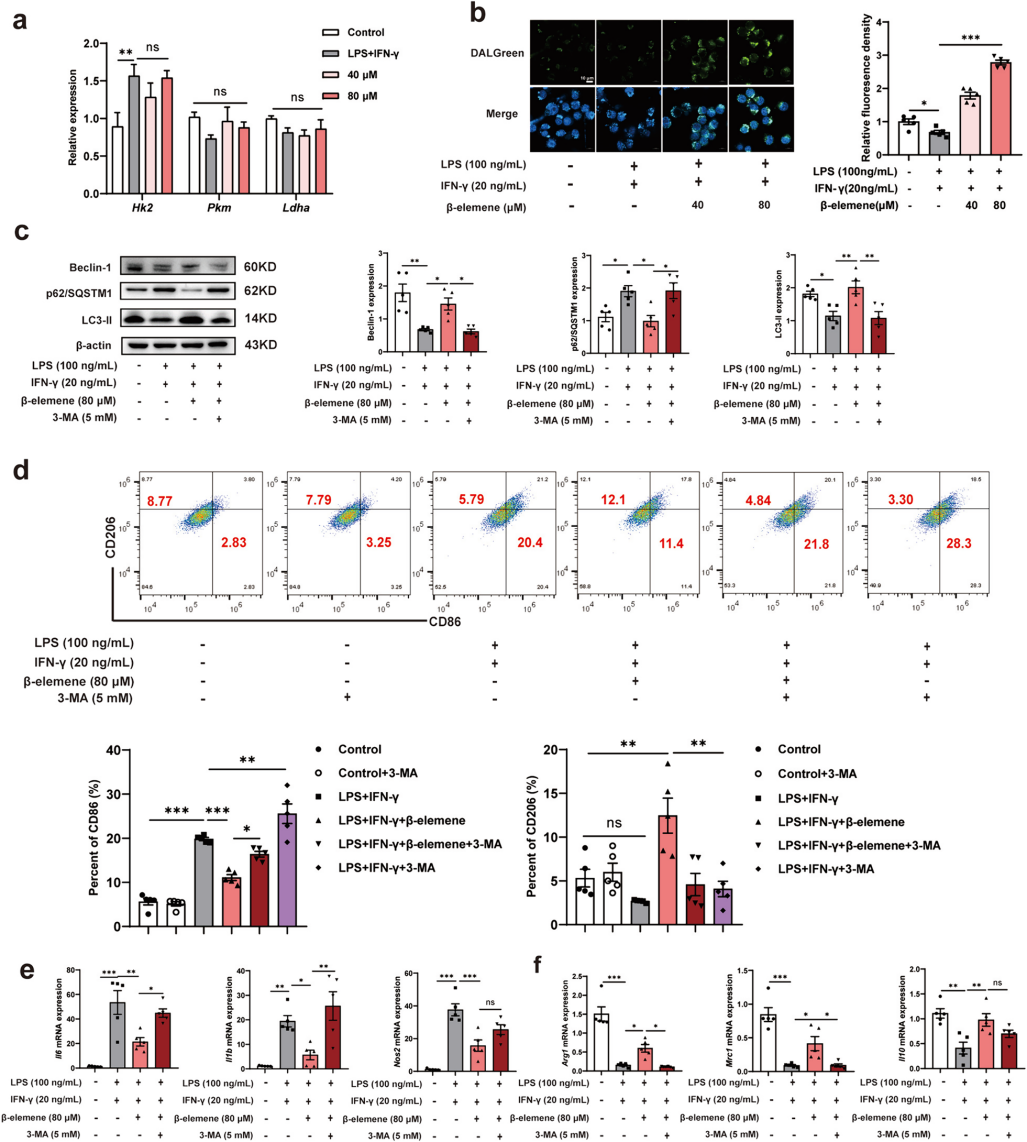
β-elemene promoted autophagy to enhance M2-like polarization of microglia. **a** The mRNA expression of the metabolic enzymes-related to glycolysis (*Hk2, Pkm, Ldha*) was examined in LPS- and IFN-γ-induced BV-2 cells; **b** autolysosomes were labled by DALGreen fluorescent dye in LPS- and IFN-γ-induced BV-2 cells, scale bar = 10 μm; **c** beclin-1, p62/SQSTM1, and LC3-II protein levels in LPS- and IFN-γ-induced BV-2 cells with indicated reagents; **d** M1/M2-like microglial proportion was analyzed in BV-2 cells by flow cytometry when treated with 3-MA; **e, f** M1/M2-like phenotype-associated gene levels. Data were represented by means ± SEM (n = 5). *p < 0.05, **p < 0.01, ***p < 0.001. *p*-values were analyzed using one-way ANOVA followed by Tukey’s test. ns, no significant difference

Thereafter, we would like to explore the other potential mechanism of β-elemene in regulating M1-like to M2-like phenotypic transformation of microglia. It has been reported that macrophages undergo the Warburg effect manifested as enhanced glycolysis when they are polarized into the M1-like phenotype. Therefore, we examined the mRNA expression of the metabolic enzymes related to glycolysis (*Hk2, Pkm, Ldha)*. As shown in Fig. 6a, β-elemene did not affect the mRNA levels of these genes in LPS-and IFN-γ-induced BV-2 cells, suggesting that the effect of β-elemene on microglia was in a glycolysis-independent manner. It has been reported that autophagy can regulate the polarization of macrophages [29, 30]. Therefore, we used DALGreen fluorescent dye to label the autophagy level in BV-2 cells. As shown in Fig. 6b, LPS- and IFN-γ stimulation decreased green fluorescence of DALGreen fluorescent dye, whereas β-elemene treatment showed increased green fluorescence, indicating enhanced autophagy. Meanwhile, LPS-and IFN-γ stimulation decreased the expression of autophagy-related protein Beclin-1 and LC3-II but increased p62/SQSTM1 expression, whereas these alterations were significantly reversed by β-elemene treatment. And the treatment of 3-MA (an autophagy inhibitor) significantly abolished the regulation of β-elemene on autophagy-associated protein expression (Fig. 6c). Then, we further clarify the relationship between autophagy and microglial polarization. As shown in Fig. 6d, compared with the control group, 3-MA alone had no effect on M1/M2-like polarization of microglia, while the addition of 3-MA increased the M1-like polarization ratio and reduced the M2-like polarization ratio of BV-2 cells under the condition of LPS and IFN-γ insult. β-elemene enhanced the transformation of BV-2 cells from M1-like to M2-like phenotype, which was abrogated with 3-MA administration (Fig. 6d). Similarly, the increased levels of M2-like phenotype-associated genes whereas decreased levels of M1-like associated genes by β-elemene were also revoked by 3-MA supplementation in LPS-and IFN-γ-treated BV-2 cells (Fig. 6e, f). These results collectively demonstrated that β-elemene promoted microglial M2-like polarization by promoting autophagy.

## β-elemene prevented the activation of AKT/mTOR signaling pathway to promote autophagy

**Fig. 7:**
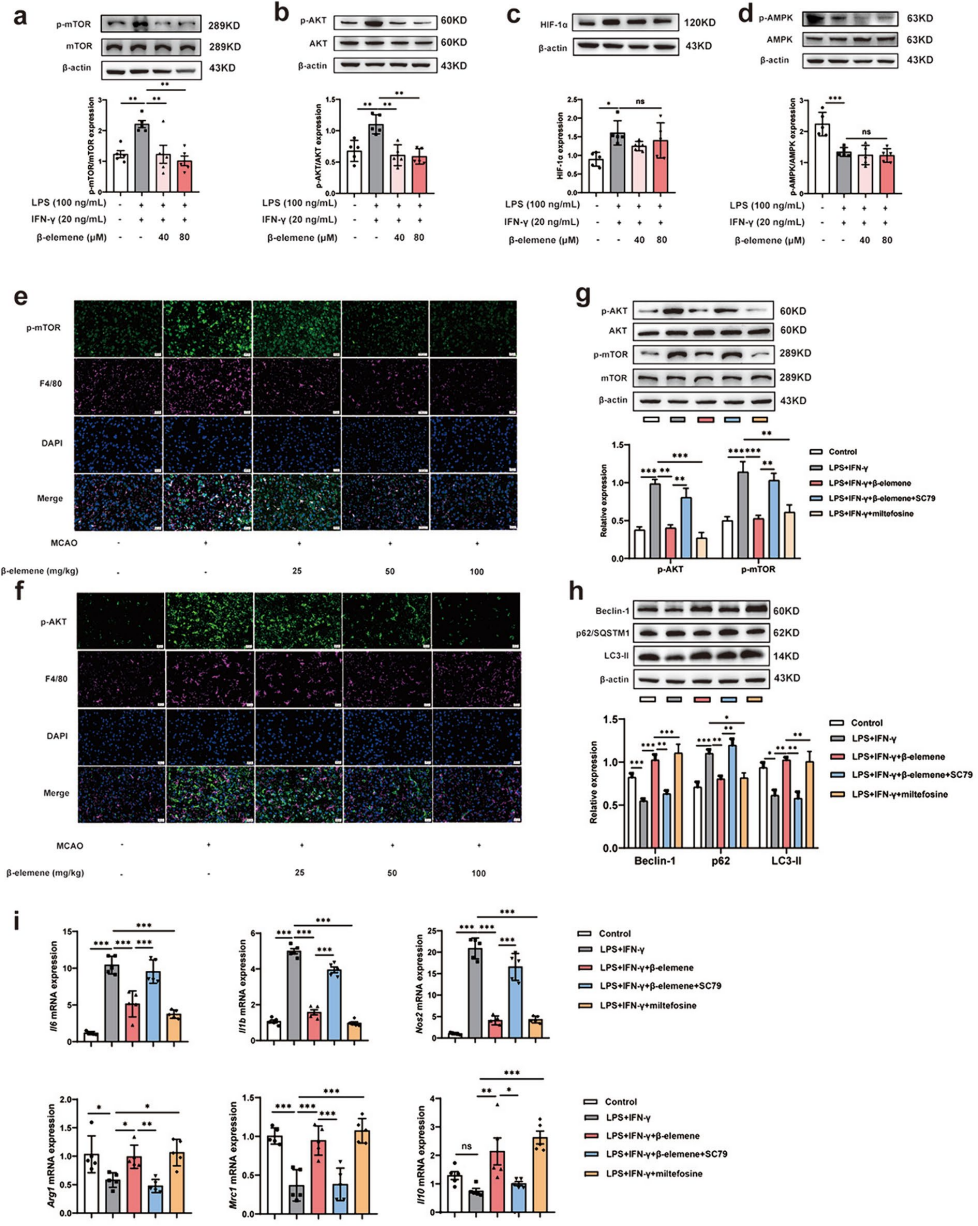
β-elemene activated autophagy through preventing the activation of AKT/mTOR signaling pathway. **a-d** p-mTOR/mTOR, p-AKT/AKT, HIF-1α and p-AMPK/AMPK protein levels were determined in LPS- and IFN-γ-induced BV-2 cells; **e, f **p-mTOR and p-AKT protein levels within microglia in the peri-infarct area of MCAO mice, scale bar = 20 μm. **g, h** p-AKT/AKT, p-mTOR/mTOR, Beclin-1, p62/SQSTM1, and LC3-II protein levels in LPS- and IFN-γ-induced BV-2 cells with indicated reagents; **i** M1/M2-like phenotype-associated gene levels; Results were indicated by means ± SEM (n = 5). *p < 0.05, **p < 0.01, ***p < 0.001. *p*-values are analyzed through one-way ANOVA followed by Tukey’s test. ns, no significant difference

Several literatures have revealed that the upstream pathway regulating autophagy is mainly involved in HIF-1α/mTOR, AMPK/mTOR, and AKT/mTOR in macrophages [31–33]. We firstly detected the phosphorylation of mTOR and found that β-elemene treatment significantly prevented the expression of p-mTOR/mTOR in LPS-and IFN-γ-induced BV-2 cells (Fig. 7a). Then, we determined the protein levels of HIF1α, p-AMPK/AMPK and p-AKT/AKT in LPS- and IFN-γ-induced BV-2 cells, and found that β-elemene significantly down-regulated the expression of p-AKT/AKT (Fig. 7b) but scarcely affected HIF-1α and p-AMPK/AMPK (Fig. 7c, d). In line with the results of in vitro studies, β-elemene also decreased the protein levels of p-AKT and p-mTOR in peri-infarct area of MCAO mice (Fig. 7e, f). To further confirm the effect of β-elemene on microglial autophagy by regulating AKT/mTOR signaling pathway, we used SC79 (AKT activator) and miltefosine (AKT inhibitor) to detect the expression of p-mTOR/mTOR and autophagy-related protein, and found that β-elemene-downregulated p-AKT/AKT and p-mTOR/TOR were significantly up-regulated under the action of SC79 in LPS- and IFN-γ-induced BV-2 cells (Fig. 7g). Similarly, increased Beclin-1 and LC3-II protein levels but reduced p62/SQSTM1 protein level of β-elemene were also significantly reversed by SC79 treatment (Fig. 7h). Subsequently, the increased mRNA levels of M2-like phenotype-associated genes but decreased mRNA levels of M1-like phenotype-associated genes by β-elemene were also revoked by SC79 supplementation in LPS-and IFN-γ-treated BV-2 cells (Fig. 7i). Miltefosine (AKT inhibitor) showed similar regulatory effects as β-elemene, further confirming the efficacy of β-elemene on autophagy by inhibiting AKT (Fig. 7g-7i). Collectively, these results revealed that β-elemene enhanced the switch of microglia from M1-like to M2-like phenotype, at least partially, through AKT/mTOR-mediated autophagy.

## Discussion

Currently, ischemic stroke is mainly treated with thrombolytic therapy and endovascular intervention, but these treatments often cause reperfusion injury [34]. Consequently, there is a high need to develop neuroprotective therapeutic strategies for combating reperfusion injury. Accumulating evidence emphasizes that post-ischemic inflammation has an enormous impact on various cerebral ischemic injury stages [35, 36]. Anti-inflammatory strategies are attractive in ischemic stroke therapy on account of their wide therapeutic window compared with the currently mainstream reperfusion-based treatments.

As the multi-functional medicine, *Curcuma wenyujin* possesses beneficial activities of enhancing blood circulation and dredging the meridians, which is adopted for treating different disorders. Over the past years, 169 compounds have been discovered from *Curcuma wenyujin* [37]. Among them, β-elemene as a volatile component can readily penetrate the BBB and has been explored extensively for treating brain cerebropathy [38, 39]. The pharmacokinetic study indicates that β-elemene distributes rapidly to the brain and the elimination is slower than in other tissues when it is intravenously administrated in rats [40]. Consequently, we investigated the effect of β-elemene on ischemic stroke injury and found that it attenuated neurological deficit, reduced the infarction volume and neuroinflammation, thus improving ischemic stroke injury in C57BL/6 J mice subjected to MCAO model and photothrombotic stroke model.

β-elemene has been explored extensively because of the extensive pharmacological effects including anti-inflammation and anti-cancer. β-elemene inactivates the miR-1323/Cbl-b/EGFR pathway to prevent the metastasis of multidrug-resistant gastric cancer cells [41]. In non-small cell lung cancer, β-elemene induces ferroptosis, resulting in the increased sensitivity of erlotinib [42]. Combination therapy with β-elemene and cetuximab remarkably inhibits the growth of colorectal cancer cells [43]. Additionally, β-elemene is also demonstrated with anti-inflammatory effect. It efficiently combats obesity-related chronic inflammation through promoting Tregs production in mice intestinal immune system [44]. β-elemene attenuates lipid-induced inflammation by down-regulating IL-6 expression in vitro [20]. Consistent with these studies, our work found that β-elemene decreased the mRNA levels of pro-inflammatory genes and increased the mRNA levels of anti-inflammatory genes in LPS- and IFN-γ-induced BV-2 cells.

Microglia are one of the first immune cell populations to react to danger signals in the central nervous system (CNS). Within minutes after ischemic stroke, microglia are activated in an M1-like phenotype and release pro-inflammatory mediators, such as IL-6, TNF-α, iNOS, ROS, leading to the break of the BBB [45]. On the contrary, activated M2-like microglia produce anti-inflammatory cytokine like IL-10, arginase 1, trophic or growth factors [46]. In spite of the dichotomy between M1/M2 polarization of microglia is a simplistic conceptual framework, it remains to be useful for enhancing our understanding of the functional status of microglia in period of injury advancement and helping us to develop novel therapy [47]. Compelling evidence has implicated that the imbalance between M1-like and M2-like microglia is responsible for the inflammation in ischemic stroke-related pathogenic mechanism [8]. Targeting microglial polarization is considered a promising therapeutic strategy for neuroinflammation. β-elemene improves M1/M2-like tumor-associated macrophage proportion and evokes a robust tumor immune response [48]. β-elemene also modulates M1-like and M2-like polarization through inhibiting MAPK signaling pathway in macrophage dysfunction resulting from high-fat diet (HFD) [28]. In our study, β-elemene favorably inactivated microglia, as evidenced by lowered Iba-1 expression, elevated branch and ending numbers per microglia. Moreover, we found that β-elemene promoted microglial polarization from M1-like to M2-like phenotype within MCAO mice and LPS- and IFN-γ-treated BV-2 cells. Furthermore, β-elemene protected neurons against OGD/R injury by inhibiting macrophages inflammatory factor release, thereby reducing neuronal apoptosis.

In our work, β-elemene significantly inhibited the TLR4/NF-κB and MAPK signaling pathways, which were consistent with previous reports [28, 49]. It has been shown that β-elemene can promote cell-protective autophagy during malignancy. In leukemia THP-1 cells, β-elemene promotes dissociation of Bcl-2/Beclin-1 complex, leading to autophagosome generation and cell autophagy [50]. Moreover, microglial autophagy has found to prevent neuronal damage by facilitating the microglial M2 polarization and decreasing its mediated inflammatory response. Enhanced autophagy inhibits NLRP3 inflammasome expression and decreases inflammatory response [51]. Inversely, autophagy inhibitor 3-MA pretreatment obviously promotes microglial activation [52]. In our study, β-elemene promoted microglial M2-like polarization both in MCAO mice and BV-2 cells. Mechanically, β-elemene prevented the activation of TLR4/NF-κΒ and MAPK signaling pathways and increased AKT/mTOR mediated-autophagy to promote microglia M2-like polarization.

Although previous researches have disclosed the anti-inflammatory effects of β-elemene, its efficacy and detailed mechanism in ischemic stroke are less reported. Our study indicated that β-elemene attenuated neurological deficit, reduced the infarction volume, and promoted the switch of microglia from M1-like to M2-like phenotype, thus improving ischemic stroke injury. Mechanically, β-elemene prevented the activation of TLR4/NF-κΒ and MAPK signaling pathways, and increased AKT/mTOR mediated-autophagy to promote microglia M2-like polarization. This study provides insight into the potential mechanism of β-elemene to be a potent agent for treating ischemic stroke injury. However, this research only demonstrated the potency of β-elemene in laboratory animal model, and further experiments remain to be needed to investigate whether β-elemene can be used in clinic for treating ischemic stroke injury.

## Conclusions

This study demonstrated the feasibility of β-elemene as a brain protection agent for treating cerebral ischemic injury. β-elemene attenuated neurological deficit, reduced the infarction volume and enhanced microglial M2 polarization in MCAO mice. Mechanically, β-elemene prevented TLR4/NF-κΒ and MAPK signaling pathway, and promoted AKT/mTOR-mediated autophagy to enhance the switch of microglia from M1-like to M2-like phenotype, thereby inhibiting inflammatory factor production to protect neurons from apoptosis. Our study implies the pharmacological action and potential mechanism of β-elemene in alleviating ischemic stroke injury, which provides some ideas for expanding the clinical application of β-elemene.

### Supplementary Information

Additional file 1.

## Data Availability

The data produced from this study are available from the corresponding author on reasonable request.

## Abbreviations

BBB: Blood–brain barrier
BSA: Bovine serum albumin
CCA: Common carotid artery
CM: Conditioned media
CNS: Central nervous system
CCK8: Cell Counting Kit-8
NBP: DL‑3‑n‑butylphthalide
DAMPs: Damage-associated molecular patterns
DMEM: Dulbecco’s modified eagle medium
ELISA: Enzyme-linked immunosorbent assay
ECA: External carotid artery
Edaravone: 3‑Methyl‑1‑phenyl‑2‑pyrazolin‑5‑one
FBS: Fetal bovine serum
H&E: Hematoxylin and eosin
IL-6: Interleukin-6
IL-1β: Interleukin-1β
ICA: Internal carotid artery
iNOS: Inducible nitric oxide synthase
LDH: Lactate dehydrogenase
MCAO: Middle cerebral artery occlusion
MCA: Middle cerebral artery
NF-κB: Nuclear factor kappa-B
OGD/R: Oxygen and glucose deprivation/reoxygenation
tPA: Tissue plasminogen activator
TNF-α: Tumor necrosis factor-α
