# Supplementary material for: β-elemene promotes microglial M2-like polarization against ischemic stroke via AKT/mTOR signaling axis-mediated autophagy
Source: Chin Med. 2024 Jun 15;19:86. doi: 10.1186/s13020-024-00946-6 (PMC11179363; doi:10.1186/s13020-024-00946-6)
Supplement: Supplementary file 1 — Additional file 1. [file 13020_2024_946_MOESM1_ESM.docx]

**β-elemene promotes microglia M2 polarization against ischemic stroke via AKT/mTOR signaling axis-mediated autophagy**

Qiong Zhao ^a^, Lu Chen ^a^, Xin Zhang ^a^, Hua Yang ^a^, Yi Li ^a,^*, Ping Li ^a,^*.

*^a^**State Key Laboratory of Natural Medicines, China Pharmaceutical University,* *#639 Longmian Dadao, Nanjing 211198, China.*

^*^Corresponding authors: China Pharmaceutical University, #639 Longmian Dadao, Nanjing 211198, China. Tel: +86 25 8327 1379; fax: +86 25 8327 1379.

Email: liping2004@126.com (P. Li); [liyi20087598@163.com](mailto:liyi20087598@163.com) (Y. Li).


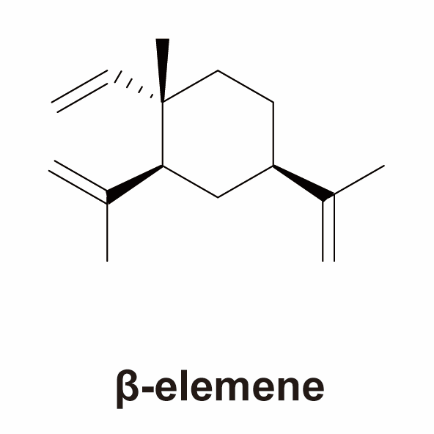


**Figure S1.** The chemical structure of β-elemene


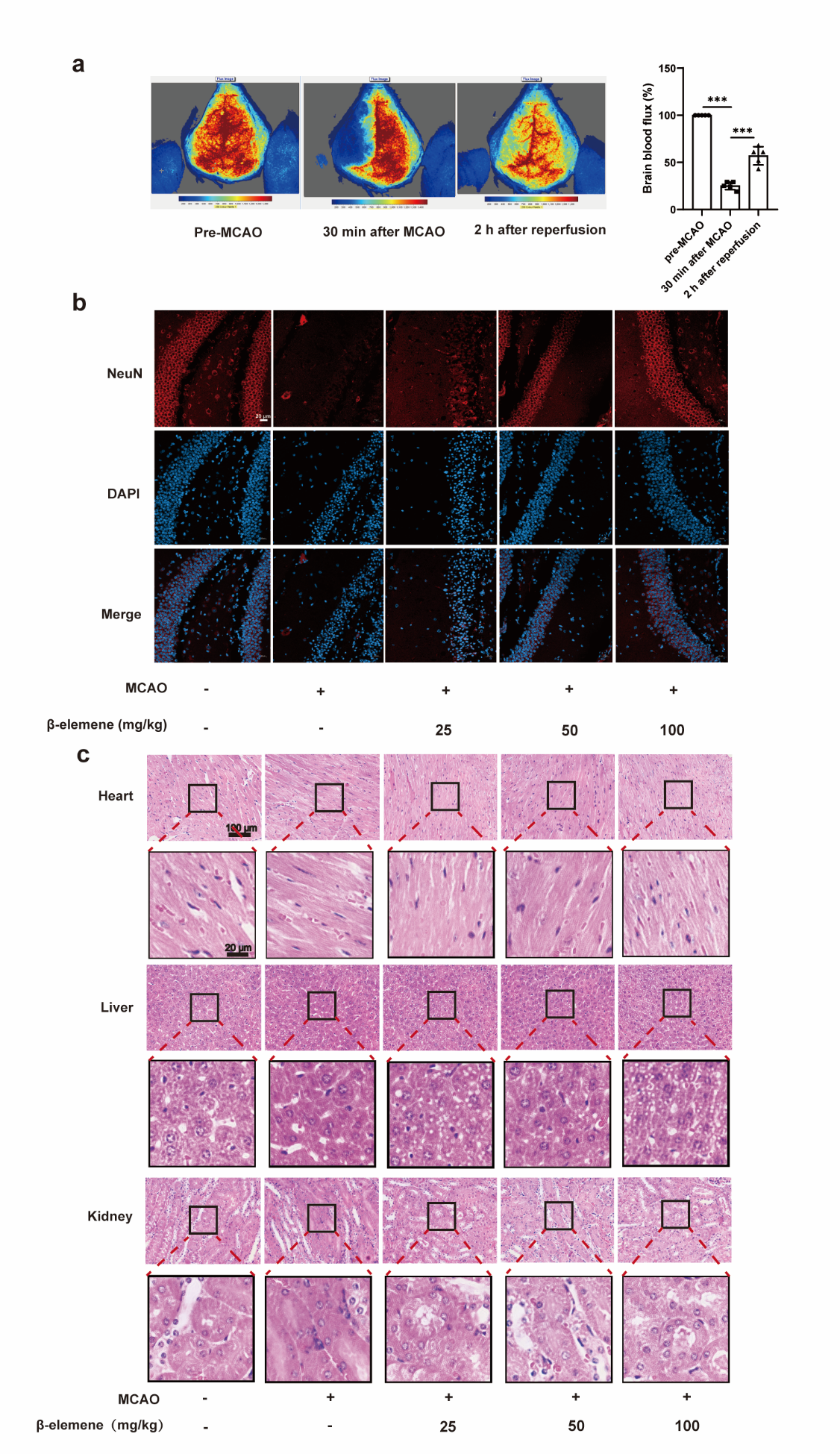


**Figure S2.** β-elemene protected the neuronal function of MCAO mice. (a) Representative LSCI (laser speckle contrast imaging) images at pre-MCAO, 30-min post-MCAO and 2-h post-reperfusion of MCAO mice; **(b)** NeuN expression within brain slices through immunofluorescence analysis. Scale bar = 100 μm; **(c)** Representative images of heart, liver, and kidney tissue of MCAO mice through H&E analysis. Scale bar = 100 μm. Results were represented by means ± SEM (n = 5). *** *p* < 0.001. *p*-values are analyzed using one-way ANOVA and Tukey’s test.


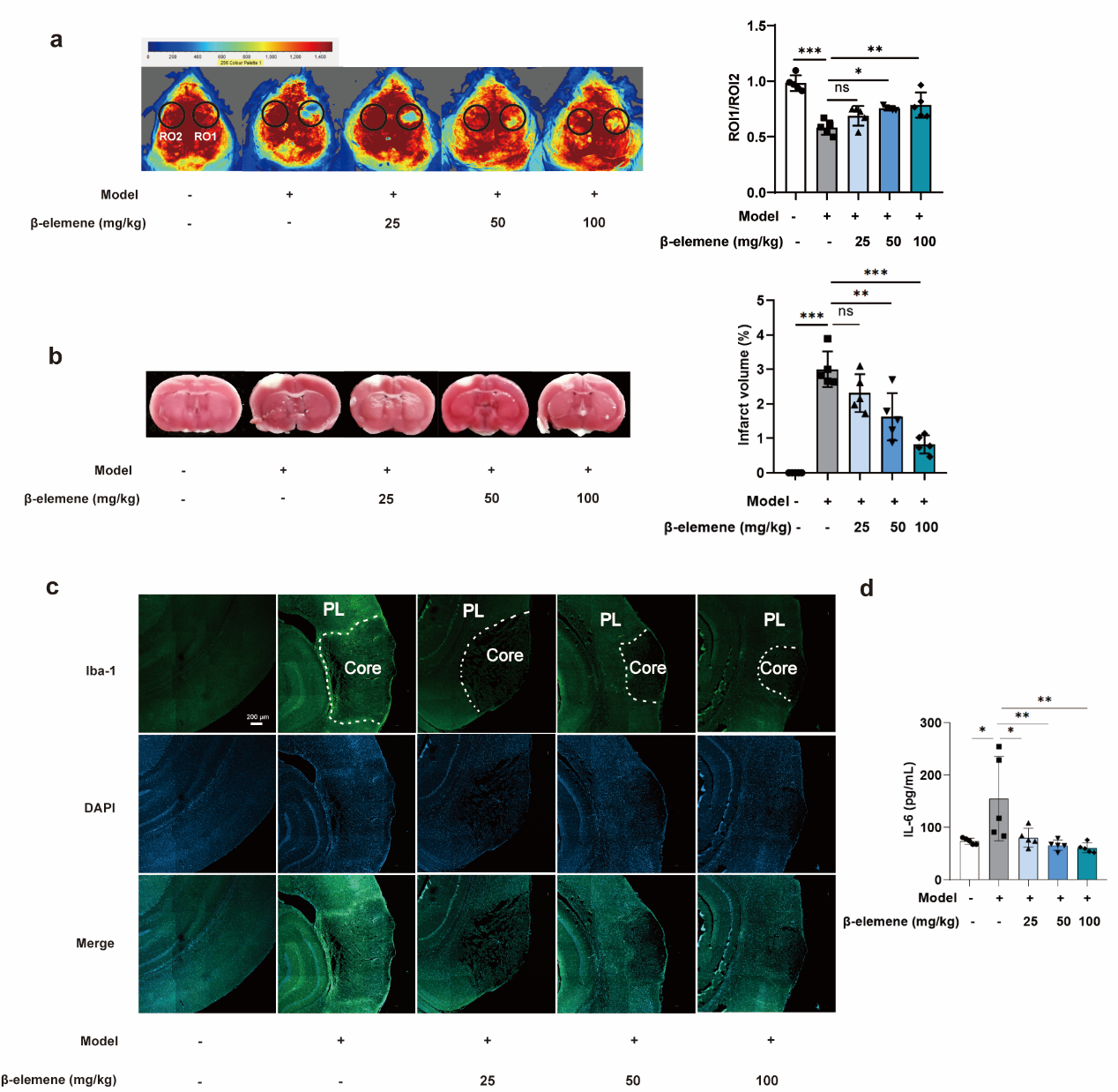


**Figure S3.** β-elemene improved cerebral ischemic injury in photothrombotic stroke model. **(a)** The CBF of cortex was detected; **(b)** The cerebral infarct volume in mice was evaluated through TTC analysis; **(c)** Microglial activation was analyzed through immunofluorescence analysis. Scale bar, 200 μm, green: Iba-1, blue: DAPI; **(d)** The level of IL-6 in serum. Results were represented by means ± SEM (n = 5). * *p* < 0.05, ** *p* < 0.01, *** *p* < 0.001. *p*-values are analyzed using one-way ANOVA and Tukey’s test.

**Table S1. Sample size, and number of deaths and excluded mice**

| **Experiments involved** | **Groups** | **Initial number** | **Number of deaths** | **Number of mice with hemorrhage or surgical accident** | **Total sample size** |
| --- | --- | --- | --- | --- | --- |
| Figure1b-1c | Sham | 5 | 0 | 0 | 5 |
|  | MCAO | 8 | 1 | 2 | 5 |
|  | 25 mg/kg | 7 | 1 | 1 | 5 |
|  | 50 mg/kg | 6 | 1 | 0 | 5 |
|  | 100 mg/kg | 6 | 0 | 1 | 5 |
|  | Butylphthalide | 7 | 1 | 0 | 6 |
|  | Edaravone | 7 | 1 | 1 | 5 |
| Figure1e-1f  Figure2a-2b  Figure5c-5d  Figure7e-7f  FigureS2b-S2c | Sham | 5 | 0 | 0 | 5 |
|  | MCAO | 7 | 1 | 1 | 5 |
|  | 25 mg/kg | 6 | 1 | 0 | 5 |
|  | 50 mg/kg | 7 | 2 | 0 | 5 |
|  | 100 mg/kg | 8 | 1 | 2 | 5 |
| Figure 1d  Figure 2e-2f | Sham | 5 | 0 | 0 | 5 |
|  | MCAO | 7 | 0 | 2 | 5 |
|  | 25 mg/kg | 6 | 1 | 0 | 5 |
|  | 50 mg/kg | 6 | 0 | 1 | 5 |
|  | 100 mg/kg | 7 | 1 | 1 | 5 |
| Figure 2c-2d | Sham | 5 | 0 | 0 | 5 |
|  | MCAO | 7 | 1 | 1 | 5 |
|  | 25 mg/kg | 6 | 1 | 0 | 5 |
|  | 50 mg/kg | 6 | 0 | 1 | 5 |
|  | 100 mg/kg | 7 | 2 | 0 | 5 |
| Figure S3a-S3b | Sham | 5 | 0 | 0 | 5 |
|  | Model | 5 | 0 | 0 | 5 |
|  | 25 mg/kg | 5 | 0 | 0 | 5 |
|  | 50 mg/kg | 5 | 0 | 0 | 5 |
|  | 100 mg/kg | 5 | 0 | 0 | 5 |
| Figure S3c-S3d | Sham | 5 | 0 | 0 | 5 |
|  | Model | 5 | 0 | 0 | 5 |
|  | 25 mg/kg | 5 | 0 | 0 | 5 |
|  | 50 mg/kg | 5 | 0 | 0 | 5 |
|  | 100 mg/kg | 5 | 0 | 0 | 5 |

**Table S2.** **Primer sequences used to detect target genes by qRT-PCR**

| **Primers** | **Sequence（5’-3’）** |
| --- | --- |
| *Il6* Forward | TACCACTTCACAAGTCGGAGGC |
| *Il6* Reverse | CTGCAAGTGCATCATCGTTGTTC |
| *Il1β* Forward | TGGACCTTCCAGGATGAGGACA |
| *Il1β* Reverse | GTTCATCTCGGAGCCTGTAGTG |
| *Actb* Forward | GGGAAATCGTGCGTGAC |
| *Actb* Reverse | AGGCTGGAAAAGAGCCT |
| *Nos2* Forward | GAGACAGGGAAGTCTGAAGCAC |
| *Nos2* Reverse | CCAGCAGTAGTTGCTCCTCTTC |
| *Arg1* Forward | CATTGGCTTGCGAGACGTAGAC |
| *Arg1* Reverse | GCTGAAGGTCTCTTCCATCACC |
| *Mrc1* Forward | GTTCACCTGGAGTGATGGTTCTC |
| *Mrc1* Reverse | AGGACATGCCAGGGTCACCTTT |
| *Il10* Forward | CGGGAAGACAATAACTGCACCC |
| *Il10* Reverse | CGGTTAGCAGTATGTTGTCCAGC |
| *Hk2* Forward | CCCTGTGAAGATGTTGCCCACT |
| *Hk2* Reverse | CCTTCGCTTGCCATTACGCACG |
| *Pkm* Forward | CAGAGAAGGTCTTCCTGGCTCA |
| *Pkm* Reverse | GCCACATCACTGCCTTCAGCAC |
| *Ldha* Forward | ACGCAGACAAGGAGCAGTGGAA |
| *Ldha* Reverse | ATGCTCTCAGCCAAGTCTGCCA |
| *Sod2* Forward | TAACGCGCAGATCATGCAGCTG |
| *Sod2* Reverse | AGGCTGAAGAGCGACCTGAGTT |
| *Cat* Forward | GGAGGCGGGAACCCAATAG |
| *Cat* Reverse | GTGTGCCATCTCGTCAGTGAA |
| *Hmox1* Forward | CACTCTGGAGATGACACCTGAG |
| *Hmox1* Reverse | GTGTTCCTCTGTCAGCATCACC |
| *Nqo1* Forward | GCCGAACACAAGAAGCTGGAAG |
| *Nqo1* Reverse | GGCAAATCCTGCTACGAGCACT |
